# Patau and Edwards Syndromes in a University Hospital: beyond palliative care

**DOI:** 10.1590/1984-0462/2024/42/2023053

**Published:** 2023-12-11

**Authors:** Ligia Marçola, Ivete Zoboli, Rita Tiziana Verardo Polastrini, Silvia Maria de Macedo Barbosa, Mário Cícero Falcão, Paula de Vicenzi Gaiolla

**Affiliations:** a Universidade de São Paulo, Faculdade de Medicina, Hospital das Clínicas, São Paulo, SP, Brazil.

**Keywords:** Trisomy 13 syndrome, Trisomy 18 syndrome, Heart defects, congenital, Palliative care, Síndrome da trissomia do 13, Síndrome da trissomia do 18, Cardiopatias congênitas, Cuidados paliativos

## Abstract

**Objective:**

To describe the newborn population with Patau (T13) and Edwards Syndrome (T18) with congenital heart diseases that stayed in the Intensive Care Unit (ICU) of a quaternary care hospital complex, regarding surgical and non-surgical medical procedures, palliative care, and outcomes.

**Methods:**

Descriptive case series conducted from January/2014 to December/2018 through analysis of records of patients with positive karyotype for T13 or T18 who stayed in the ICU of a quaternary hospital. Descriptive statistics analysis was applied.

**Results:**

33 records of eligible patients were identified: 27 with T18 (82%), and 6 T13 (18%); 64% female and 36% male. Eight were preterm infants with gestational age between 30–36 weeks (24%), and only 4 among the 33 infants had a birth weight >2500 g (12%). Four patients underwent heart surgery and one of them died. Intrahospital mortality was 83% for T13, and 59% for T18. The majority had other malformations and underwent other surgical procedures. Palliative care was offered to 54% of the patients. The median hospitalization time for T18 and T13 was 29 days (range: 2–304) and 25 days (13–58), respectively.

**Conclusions:**

Patients with T13 and T18 have high morbidity and mortality, and long hospital and ICU stays. Multicentric studies are needed to allow the analysis of important aspects for creating protocols that, seeking therapeutic proportionality, may bring better quality of life for patients and their families.

## RESUMO

**Objetivo:**

Descrever a população de recém-nascidos com síndrome de Patau (T13) e Edwards (T18) portadores de cardiopatias congênitas, que permaneceram em Unidades de Terapia Intensiva (UTI) de um complexo hospitalar quaternário, com relação a conduta cirúrgica ou não, cuidados paliativos e seus desfechos.

**Métodos:**

Série de casos de pacientes internados entre janeiro de 2014 a dezembro de 2018, com análise dos prontuários de portadores de T13 ou T18 que permaneceram internados em UTI que recebem neonatos nesse hospital quaternário. Utilizou-se análise estatística descritiva.

**Resultados:**

Foram identificados 33 prontuários para análise — 27 T18 (81,8%) e seis T13 (18,2%); 64% do sexo feminino e 36% do sexo masculino. Oito foram prematuros, nascidos com 30 a 36 semanas (24,2%), e apenas quatro nasceram com mais de 2500 g (12,1%). Quatro pacientes foram submetidos a cirurgia cardíaca e um deles foi a óbito. A mortalidade intra-hospitalar foi de 83% para T13 e 59% para T18. A maioria apresentava outras malformações e foi submetida a outras cirurgias. Cuidados paliativos foram oferecidos a 54% dos pacientes. A mediana do tempo de hospitalização para T18 e T13 foi respectivamente de 29 dias (variação: 2–304) e 25 dias (13–58).

**Conclusões:**

Pacientes com T13 e T18 cursam com alta morbimortalidade e longa permanência hospitalar em UTI. São necessários estudos multicêntricos para melhor análise de aspectos importantes para a criação de protocolos que, buscando proporcionalidade terapêutica, tragam melhor qualidade de vida para os pacientes e suas famílias.

## INTRODUCTION

Edwards Syndrome — trisomy of chromosome 18 (T18) — and Patau Syndrome — trisomy of chromosome 13 (T13) — are, respectively, the second^1-3^ and third^1,3^ most prevalent aneuploidies. Both have high morbimortality rates and neurologic development delay, with severe cognitive and motor alterations.^4^ More than 80% of the cases may present heart diseases such as abnormal septum and patent Ductus Arteriosus (PDA), among other more complex conditions.^2,4,5^

The overall survival of children with these syndromes is less than 15 days;^3,5-7^ and the majority die before the first year of life.^3,6,7^ These syndromes used to be described as lethal, and exclusive palliative care was the only possible treatment.^2,4^ However, this has been progressively changing.^2,3,8^ Invasive procedures like tracheostomy and gastrostomy tube placement have increased over time, as well as surgeries indications.^2^ Studies have shown an increase in survival rates beyond the second year of life; 50 to 60% of the individuals that survive for more than six months live for more than ten years, especially when mosaics and partial trisomies are involved^6^ and when heart abnormalities are surgically corrected.^1,3^ Thus, palliative care may no longer be the only treatment, but otherwise associated with curative treatment, matching it to families’ goals and patients’ needs.^7,8^

On the one hand, palliative or correction surgery for a congenital heart disease in children with T13 or T18 can allow families to take their kids home, which is the goal for many of them.^3,5,8^ On the other hand, studies have shown there is indeed a higher mortality rate of infants with T13 and T18 than in the non-syndromic population that underwent surgery,^1,3,5^ in addition to the complications and other comorbidities presented after surgical procedure.^1,6,7^ Other ethical questions such as quality of life after surgery, potential of providing unrealistic expectations for the families and risk of improper allocation of time and resources must also be considered.^4^

Thus, it is important to know the real current situation regarding the number of patients with those syndromes who are undergoing surgeries, their outcomes, and factors that influence postoperative prognosis, so that guidelines may be designed to help health care teams and families to make treatment decisions.^1,8^

The objective of the current study was to describe the population with T13 and T18 with congenital heart diseases admitted in an Intensive Care Unit (ICU) of a quaternary care hospital complex and to analyze those submitted or not to surgical or non-surgical medical procedures and their outcomes — death, hospital discharge or transference to another hospital.

## METHOD

The study was performed at a quaternary university hospital in the city of São Paulo, in the three ICUs where newborns are admitted: Neonatal Intensive Care Unit (NICU)-1 — which admits only inborn neonates from the maternity ward annex to the hospital; NICU-2 — a national reference external nursery that admits outborn neonates and infants within 45 days of life from the entire hospital complex itself and other services; and PPNICU (Preoperatory Pediatric and Neonatal Intensive Care Unit of the Heart Institute).

This is a descriptive series of cases in which, after approval from the Ethics and Research Committee of the hospital (protocol number 3.618.578), all positive karyotype results for T13 and T18 from January 1^st^, 2014 to December 31^st^, 2018 were collected from the hospital’s laboratory records and, based on them, the corresponding medical records were analyzed. In some cases, only the test was performed, but there was no follow-up of the patient in the service. Therefore, inclusion criteria were records of newborns with T18 and T13 (with diagnosis confirmed by karyotype) with congenital heart disease admitted in at least one of the three ICU of the study in the study period. Exclusion criteria were records of newborns with T18 or T13 without congenital heart disease, with no karyotype confirmation, incomplete records or patients not followed-up at the Institution or admitted to other wards.

The variables studied were: age on admission in the ICU; gestational age (weeks), birth weight (g) and Apgar Score; syndromic diagnosis; time to karyotype result; presence and type of cardiopathy; mother’s age and comorbidities; prenatal care; whether prenatal diagnosis of the syndrome and cardiopathy had been made; comorbidities (pulmonary hypertension, renal failure, liver dysfunction or lesion, neuropathy); other malformations; surgeries (cardiac and/or others) and age on the date of surgical interventions; duration of invasive ventilatory support; other invasive interventions (dialysis, vasoactive drugs, central venous catheter, parenteral nutrition, vesical catheterization, non-invasive ventilation, transfusion of blood components); palliative care (defined in this study as all treatments and care with the objective of providing well-being and comfort to the patients and their family); outcomes and, in case of death, the cause; time of hospitalization and health condition at discharge (tracheostomy, gastrostomy, nasogastric tube, oxygen dependence, anticonvulsant drugs, drugs for heart failure treatment).

Descriptive statistical analysis was done using Microsoft Excel^®^. The number of records found didn’t allow further analysis or comparisons.

## RESULTS

**Figure 1.**
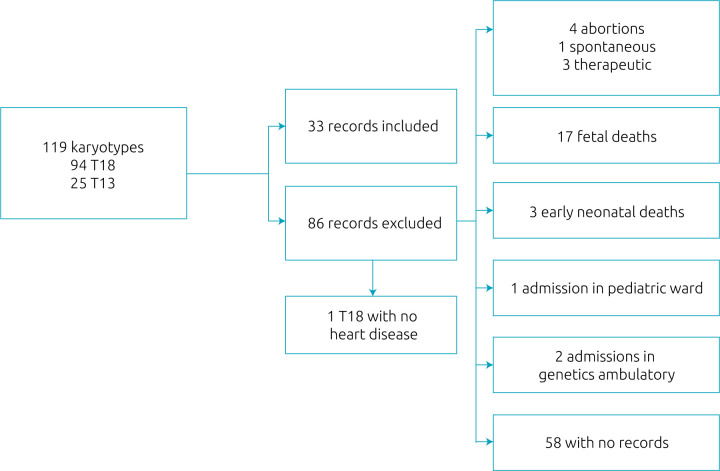
Diagram shows total karyotypes selected, total cases included (Trisomy 13 and Trisomy 18) and a list of excluded cases

T18: Trisomy 18; T13: Trisomy 13

Karyotypes analysis from the hospital’s laboratory records is shown in Figure 1. After applying the inclusion and exclusion criteria, 33 eligible records were identified (28% of all karyotypes analyzed at the laboratory in the study period) with T13 or T18: 27 T18 (82%), and 6 T13 (18%); 21 female (64%) and 12 male (36%). Table 1 shows characteristics of the patients and their mothers, as well as their prenatal care. For the cases without prenatal diagnosis of the syndrome, the average time for karyotype result was 8.4 days from the day of collection, ranging from 3 to 30 days. 

**Table 1.** Characteristics of Trisomy 13 and Trisomy 18 patients.

	n	%
Gestational age at birth (weeks)		
<30	0	0
30–36	8	24.2
>36	25	75.8
Birth weight (g)		
<1000	0	0
1000–1499	6	18.2
1500–2500	23	69.7
>2500	4	12.1
Apgar score at the 5^th^ minute <7		
Yes	5	15.1
No	28	84.9
Age on admission		
At birth	26	78.8
Until 28 days	5	15.1
>28 days	2	6.1
Mother’s age (years)		
<18	1	3
18–34	19	57.6
>34	13	39.4
Presence of maternal comorbidity	12	36.4
Prenatal care performed	33	100
Prenatal diagnosis		
Syndrome	10	30.3
Heart disease	23	69.7

Six cases of patients with T13 were studied. All of them had other associated malformations (Dandy Walker, omphaloceles, cleft palate), and only one had no comorbidities. The only one who did not die was a mosaic patient for T13 who had an Atrial Septal Defect (ASD) and was discharged with oral feeding after the surgical correction of omphalocele. This happened after 58 days of hospitalization, 29 days of mechanical ventilation and other invasive interventions. Regarding heart diseases, 1 patient had Tetralogy of Fallot and the other five had PDA, ASD and/or Ventricular Septal Defect (VSD), as well as heart valve dysplasia. None of them underwent surgical correction procedures. Of the five patients with T13 who died, three had cardiac causes. Regarding the other two patients, in one of the cases the death certificate was unreadable and in the other one, the cause of death was unknown, as it occurred in another hospital where the newborn had been transferred to. Among these 5 patients that died, three did not receive mechanical ventilation and two of them did not receive any intervention during ICU stay. The median length of stay in the ICU for these 5 patients was 23 days, ranging from 13 to 34 days. All of them received palliative care. 

Regarding T18, 27 cases were studied. Table 2 shows the characteristics and interventions related to these 27 patients and their outcomes can be seen in Table 3. Among these patients, there was only one mosaic with pulmonary stenosis, esophageal atresia, and renal malformation. The patient was discharged with home oxygen therapy and medication for congestive heart failure after 127 days of hospital stay and after the correction of esophageal atresia and gastrostomy. The patient received mechanical ventilation for 27 days and underwent other invasive interventions. Ten patients with T18 were discharged home. Of these, three underwent heart surgery and all of them underwent other surgeries (correction of malformations and gastrostomies), mechanical ventilation and other invasive interventions during hospital stay. The median duration of mechanical ventilation among these 10 patients was 9 days, ranging from 1 to 71 days. Death occurred in 16 patients with T18: three had complex heart malformations and the other 13 had other heart defects such as ASD, VSD, PDA, and valve dysplasia. The 6 very low birth weight infants were part of the 16 deaths of T18 patients and morbidities associated to preterm birth were related to their death. All patients with T18 who died received invasive interventions; six underwent non-cardiac surgeries (malformation correction, gastrostomy, surgical treatment of prematurity complications); six received mechanical ventilation by tracheal tube and five received non-invasive ventilation — the other five were not ventilated, only receiving oxygen therapy via intranasal catheter. 

**Table 2.** Characteristics and interventions of carriers of trisomy 18.

	Yes		No	
	n	%	n	%
Comorbidities	22	81.5	5	18.5
Other malformations	22	81.5	5	18.5
Heart disease surgeries	4	15	23	85
Other surgeries	16	59.2	11	41.8
Invasive interventions	27	100	0	0

**Table 3.** Outcomes of carriers of Trisomy 18.

	n	%	Days of hospitalization (median and limits)
Death	16	59.3	14 (2–113)
Heart cause	9	56	
Non-heart causes	7	44	
Transfer to origin	1	3.7	22
Discharge	10	37	68 (29–304)
Oxygen dependence	7	70	
Drugs for heart failure	8	80	
Tracheostomy	2	20	
Gastrostomy	10	100	
Anticonvulsant drugs	2	20	

Only 4 (12%) of the 33 studied patients underwent heart surgery during hospital stay, all of them with T18. Surgeries and their outcomes are described on Table 4.

**Table 4.** Heart surgeries in Trisomy 18 patients.

Case	Surgery performed	Age (days)	Outcome	Days in hospital
1	PDA closure and pulmonary artery banding	83	Discharged	101
2	Pulmonary artery banding	47	Death due to sepsis	80
3	Correction of interatrial communication, interventricular communication, and PDA	41	Discharged	150
4*	Correction of Atrial Septal Defect and Ventricular Septal Defect	395	Discharged	120

* The family came by themselves from Bahia to São Paulo, with a 6-month-old baby, with no diagnosis of trisomy 18. After the diagnosis, the patient was followed-up in the outpatient clinic. PDA: Patent Ductus Arteriosus.

Palliative care was offered to 18(54%) out of 33 patients and 3 of these 18 (17%) were discharged from ICU. Considering patients that died, 75% received palliative care.

## DISCUSSION

In five years, we found 33 patients with T13 or T18 who were admitted in the hospital complex. These results only allowed a descriptive statistical analysis. Although the study hospital is a referral center for Fetal Medicine, this number was expected, considering the rarity of these syndromes (T18: 1.07 and T13: 0.81, per 10,000 live births).^1,9^ Other studies carried out in single centers also found relatively small numbers: in a Japanese center, 17 patients with T18 who underwent surgeries in 5 years were studied;^10^ in an American center, records of 19 patients with T18 or T13 and congenital heart diseases who underwent surgeries were found in 20 years.^5^ In countries where abortion of fetus with those genetic conditions is common, the number of patients available to study is even lower. In Portugal, there were 6 cases in 25 years of research.^9^

The analysis of the characteristics of patients and their mothers, seen in Table 1 — predominance of females, advanced maternal age (as compared to the general population), intrauterine growth restriction and low birthweight, low vitality at birth, prevalence of other malformations — is in accordance with the literature.^9,11,12^ Regarding prenatal diagnosis, however, detection rates of the syndrome (30.3%) and cardiac malformations (69.7%) are below average of developed countries,^12^ which points to the need for better prenatal care available to plan the birth of these children in proper places and with proper staff.

Few heart surgeries were performed, despite the majority of study subjects having simple heart diseases eligible for palliative care and/or correction. Among the patients who underwent these surgeries, there was only one death, unrelated to heart diseases or to the surgery itself (sepsis). The literature shows a higher rate of complications^7^ and mortality among patients with T18 and T13 that underwent heart diseases than patients without these syndromes, but still with lower rates than those classically expected. On the other hand, surgeries are also responsible for the longer lifespan of survivors.^1,6,13^

Patients with T13 had the most severe conditions and worse prognosis — the only one who survived was a mosaic. Also, patients born with less than 1500 g had a bad prognosis — all of them died. A large study from 2014 with very low birth weight infants by the American Newborn Research Network showed that, on average, T13 neonates survived for 1 day and T18 for 5 days. In this cohort of 161 infants with birth weight <1500 g, eight underwent surgeries and two of them were discharged home, with a discharge rate of 11% for T13 and 9% for T18. These results were related to a limitation of therapeutic efforts, in addition to the higher prevalence of prematurity complications.^12^

Back to the present study, prolonged hospital admissions were observed, involving many invasive procedures and surgeries in most of the cases; among the patients who were discharged, the majority went home using more than one assistive device, along with many medications. However, drawing a parallel with extremely preterm infants, a study published in 2021 that enrolled neonates from the Korean Neonatal Network with birthweight <500 g showed that, among patients who were discharged home, the median hospital length of stay before discharge was 132 days (range: 69–291). Many of the infants also underwent surgeries and 53% of them were discharged with assistive devices.^14^ In Canada, infants born at a gestational age of 23–28 weeks had a median hospital length of stay of 41 days (range: 1–77) and, among the discharged ones, the median hospital length of stay was 61 days (range: 34–90).^15^

Regarding palliative care, it has been increasingly offered at the ICUs of the study hospital, and it was noticeably present among the most severe patients, who died. Many infants who received palliative care were discharged home, showing the importance of this kind of assistance in an early stage and not only at the end of life and, also, that the idea that Pediatric Palliative Care (PPC) is only for dying children and shortens life is only a myth.^9,16^ PPC is a specialized medical care that should be indicated as soon as a life-limiting condition is diagnosed, whether there is availability or not of curative treatment.

In the case of patients like the ones of the study, indications for surgery as early as possible must be considered, especially for children with the best clinical conditions, with simple congenital heart diseases^1,2,5^ and whose families aim for care outside the hospital — patients in these conditions are not rare.^3,5,8^ Many children are followed in PPC ambulatories after discharge, for continuous symptom care and discussions regarding course of treatment and, if the time comes, to prepare for end-of-life.^16^ Studies should be designed to evaluate the performance of palliative cardiac surgeries, in order to remove these children from the hospital, bringing more comfort to the family and reducing hospital costs, since the vast majority of these patients have prolonged hospitalizations, mostly in ICUs (neonatal and pediatric).

Questions and participation of the society regarding this discussion are increasing. Regarding T18, there are movements of families of children, youth, and adults with Edward’s Syndrome in Brazil, like *T18 Brasil*. Recently, data collected by this association showed that, from September 2008 to March 2022, 1643 people were registered with suspected or diagnosed T18 in the country. Among those, 295 are still alive (18%). These numbers can be even higher, since 13% of the registers are incomplete and do not have this information. Also, in this registry, 312 (19%) patients live or have lived for more than 1 year and, of those, 46 have lived for more than 10 years with T18.^17^ Although these are preliminary data from a study with limitations that depend on the register made by the family, when there is an intention to join the association and, thus, with possible selection bias, this is the most concrete Brazilian numbers related to these genetic conditions. They show significant prevalence and the social importance of these individuals and their families. 

In conclusion, patients with T13 and T18 have high morbidity and mortality, in addition to long hospital stays, mostly in ICU. The study of these syndromes in Brazil and other countries is extremely relevant for the families and health care services involved. It is necessary to perform multicenter studies that describe the prevalence and the clinical evolution of carriers of these syndromes — a higher number of cases would allow further analysis of important aspects that would help to better design guidelines for adequate care of these infants. These syndromes are severe conditions with a relevant number of affected individuals, so defining therapeutic proportionality correctly should increase the quality of life of individuals and their families.
